# LINC01128 expedites cervical cancer progression by regulating miR-383-5p/SFN axis

**DOI:** 10.1186/s12885-019-6326-5

**Published:** 2019-11-28

**Authors:** Yi Hu, Yan Ma, Jie Liu, Yanlin Cai, Mengmeng Zhang, Xiaoling Fang

**Affiliations:** 1 Department of Obstetrics and Gynaecology, The Second Xiangya Hospital, Central South University, 139 Renmin road, Changsha, 410011 Hunan China; 2 Department of Obstetrics and Gynaecology, the First Affiliated Hospital of University of South China, Hengyang, 421001 Hunan China

**Keywords:** LINC01128, miR-383-5p, SFN, Cervical cancer

## Abstract

**Background:**

Cervical cancer (CC), causing significant morbidity and mortality worldwide, is one of the most common gynecological malignancies in women. SFN has been reported as a potential prognostic marker with apparent high expression in tumors. Nevertheless, the function mechanism of SFN is not clear yet in CC.

**Methods:**

The relative expressions of RNAs were detected by real-time quantitative PCR (RT-qPCR). Colony formation assay, EdU stained assay and CCK-8 assay were to check cell proliferation ability in CC. Flow cytometry and apoptosis related proteins analysis were used to measure cells apoptosis capacity. Luciferase reporter assay and RNA pull down assay were to verify the molecular mechanism.

**Results:**

SFN was highly expressed in CC tissues and CC cell lines compared with normal tissues and normal cell line. After interfering SFN, cell proliferation, migration and invasion ability was inhibited as well as cell apoptosis ability was promoted. In subsequence, miR-383-5p exhibited conspicuous low expression in CC tissues. And miR-383-5p was found to bind to SFN and have anti-cancerous effects in CC. Moreover, LINC01128 displayed remarkable high expression in CC tissues. Besides, LINC01128 shortage could reduce the expression of SFN at mRNA and protein levels. And the affinity between LINC01128 and miR-383-5p was verified. In the end, it was proved that LINC01128 could enhance cell proliferation, migration and invasion as well as inhibit cell apoptosis by binding with miR-383-5p and upregulating SFN.

**Conclusion:**

LINC01128 expedited cells cellular process in CC by binding with miR-383-5p to release SFN.

**Graphical Abstract:**

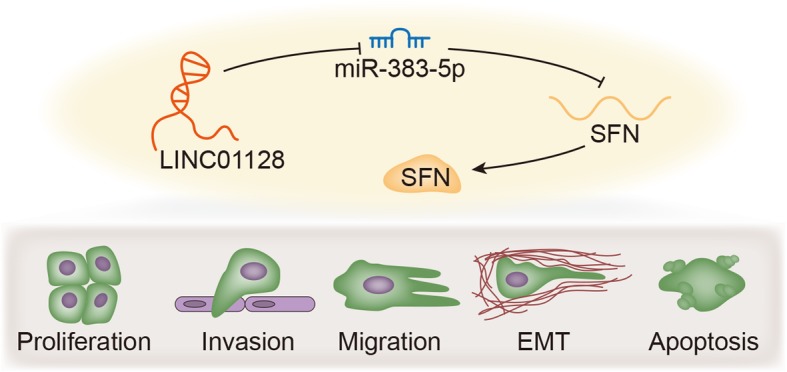

## Background

Cervical cancer (CC) is the second leading cause of cancer-related deaths in women worldwide due to its high incidence and mortality [1, 2]. Although the treatment strategies for CC have made great progress, such as surgical resection, radiotherapy and chemotherapy, the long-term prognosis of patients with cervical cancer is still not satisfactory due to frequent postoperative recurrence and resistance to radiotherapy and chemotherapy [3]. In recent years, molecular targeted therapies have significantly advanced the prognosis of many cancers, such as melanoma, breast, lung and prostate cancer [4, 5]. Nevertheless, at present, targeted therapy for the molecular mechanism of CC has not been promoted and popularized in the worldwide. Hence, further understanding of the molecular mechanism of the occurrence and development of CC will contribute to the development of more effective CC treatment.

Stratifin (SFN) has been reported to enhance lung adenocarcinoma development at an early stage [6] and correlate with poor prognosis of ovarian cancer patients [7]. Moreover, low expression of SFN has also been found to indicate poor survival of esophageal squamous cell carcinoma sufferers [8]. However, the role of SFN in CC was rarely mentioned. It has been well-established that microRNAs (miRNAs) induce translation inhibition or promotion of their target messenger RNA (mRNA) through base pairs at partial or complete complementary sites [9–12]. Nowadays, related biological research indicated the important role of non-coding RNA in many cancers [13–15]. Among them, long non-coding RNAs (lncRNA) play crucial roles in a host of biological processes. And lncRNAs are capable of regulating the expression of genes in various biological functions [16]. Among these non-coding RNAs, miRNAs and lncRNAs belong to two major groups. miRNAs are short RNAs that have a length of 21–25 nucleotides, whereas lncRNAs are long-stranded RNAs that have a length of 200 nucleotides [17–20]. It has been confirmed that one of the mechanisms of lncRNAs is the physical binding of miRNAs to reduce the inhibition of miRNAs on their true target mRNAs [21–23]. Therefore, these lncRNAs are referred to as endogenous competitive RNAs (ceRNA) [24, 25]. Hence, we would search for the lncRNA that could free SFN form the regulation of its upstream miRNA.

Taken together, the purpose of this study was to explore the expression and action of SFN, and elucidate the action mechanism of SFN in CC in detail.

## Methods

### Patients and tissue samples

Between 2013 and 2018, 33 pairs of cervical cancer tissues and adjacent normal tissues were collected from patients who underwent surgery at The Second Xiangya Hospital, Central South University. What’s more, none of the patients in this study received chemotherapy or radiotherapy before surgery. Tissue specimens were frozen in liquid nitrogen at − 80 °C at the time of collection.

### Cell culture

Cervical cancer cells (HeLa, SiHa, CaSki and C33a) and normal cervical cell (Ect1-E6E7) were purchased from the Cell Culture Bank of the Chinese Academy of Sciences (Shanghai, China). Cells were all cultured in DMEM (Thermo Fisher Scientific, Waltham, MA, USA) containing 10% fetal bovine serum (FBS; Thermo Fisher Scientific). Cells were cultured with 5% CO_2_ at 37 °C.

### Cell transfection

HeLa or SiHa cells were plated in 6-well plates, and then incubated for one day. After that, the specific short hairpin RNAs (shRNAs) of SFN (sh-SFN-1/2) or LINC01128 (sh-LINC01128–1/2) and their negative controls (sh-NCs), along with mimics and inhibitor of miR-383-5p or miR-107-mimics, NC mimics/inhibitor were purchased from Genepharma (Shanghai, China). The above plasmids were separately transfected into HeLa or SiHa cells via Lipofectamine 2000 (Invitrogen, Carlsbad, CA, USA) after 48 h.

### RNA extraction and real-time quantitative PCR (RT-qPCR)

Tissues and cells were lysed via Trizol reagent (Invitrogen), and then total RNA was extracted and reverse transcribed into cDNA via a Reverse Transcription Kit (Invitrogen). Then, RT-qPCR was conducted through SYBR Green RT-PCR Kit (Invitrogen) on an Applied Biosystems 7300 (Thermo Fisher Scientific). Results were calculated via the 2^−ΔΔCt^ method. GAPDH/U6 level was measured as the internal control.

### Cell counting kit-8 (CCK-8) and Colony formation assays

The number of HeLa or SiHa cells was 1000, which was seeded in 96-well plates and incubated under the standard conditions. CCK-8 solution (Dojindo, Tokyo, Japan) was added to each well. Then, cells were incubated for another 4 h. The absorbance at 450 nm was evaluated through a microplate reader (Olympus, Tokyo, Japan). For colony formation assay, HeLa or SiHa cells in medium (Thermo Fisher Scientific) were seeded in 6-well plates and incubated for two weeks. Colonies (≥50 cells) were formed and then manually counted.

### 5-ethynyl-2′-deoxyuridine (EdU) incorporation assay

EdU labeling medium was added to cell culture with EdU labeling kit (RiboBio, Guangzhou, China). HeLa or SiHa cells after transfection were incubated for 2 h, and then fixed with 4% paraformaldehyde (Sigma-Aldrich, St. Louis, MO, USA). Cells were washed at room temperature by PBS (Sigma-Aldrich) dyed with anti-EdU (Acbam, Cambridge, USA) working solution (Invitrogen). Under the same conditions, cells were again washed with Triton X-100 (Solarbio, Shanghai, China) in PBS (Sigma-Aldrich). Cells were observed via a fluorescent microscopy (Leica, Wetzlar, Germany).

### Northern blot

This experiment was performed as previously described [26].

#### Western blot

Transfected HeLa or SiHa cells were isolated via RIPA lysis buffer (Invitrogen). Then, the BCA (Invitrogen) was employed to evaluate the concentration of protein. Proteins were separated by SDS-PAGE (Millipore, MA, USA), followed by transferred into PVDF membranes (Millipore). After being blocked with 5% nonfat milk, membranes were incubated overnight with primary antibodies at 4 °C: anti-Bax (ab32503, Abcam), anti-Bcl-2 (ab182858, Abcam), anti-SFN (ab14123, Abcam) anti-E-cadherin (ab194982), anti-N-cadherin (ab202030), anti-Vimentin (ab193555), anti-ZEB1 (ab228986), anti-Slug (ab51772), anti-Twist (ab187008), anti-Snail (ab229701) and anti-GAPDH (ab8245, Abcam), then cultivation at room temperature with secondary antibody for 1 h. GAPDH was employed as the internal parameter. Finally, the ECL Kit (Thermo Fisher Scientific) was selected to visualize protein bands.

### Flow Cytometry

Transfected HeLa or SiHa cells were cultivated into 6-well plates. After being washed twice with cold PBS (Sigma-Aldrich) and re-suspended, cells were fixed through ice-cold ethanol (Sigma-Aldrich). Finally, flow cytometer (BD Biosciences, San Diego, CA, USA) was employed to analyze cell apoptosis.

### Transwell assay

The re-suspended cells in serum-free medium were seeded into the upper chamber, and the basolateral chamber was filled with 10% PBS. Transwell chambers pre-coated with or without Matrigel (Millipore, Massachusetts, USA) were employed to carry out the invasion or migration assay. An inverted microscope (Carl Zeiss, Jena, Germany) was applied to observe the migrated or invaded cells.

### Sphere formation assay

HeLa or SiHa cells were harvested, counted and incubated in 6-well ultra-low attachment plates (Corning Incorporated, Corning, NY, USA) in serum-free RPMI 1640 medium (Invitrogen, Shanghai, China) to form sphere. The RPMI 1640 medium is supplemented with 20 ng/ml human FGF (Gibco, Thermo Fisher Scientific, Waltham, MA, USA), 20 ng/ml human EGF (Gibco), 1% N2 supplement (Gibco) and 1% B27 (Gibco). 2 weeks later, the number of spheres was counted by a light microscope (Nikon Corporation) and the number was recorded.

### Subcellular fractionation

Isolation and purification of cytoplasmic and nuclear RNA were conducted via a Cytoplasmic and Nuclear RNA Purification Kit from Norgen (Ontario, Canada). The expression levels of LINC01128, GAPDH and U6 were separately evaluated via RT-qPCR analysis.

### RNA pull-down assay

miR-383-5p biotin probe and miR-383-5p no-biotin probe were treated with M-280 Streptavidin magnetic beads (Invitrogen) to generate probe-coated beads. Subsequently, cells were collected and dissolved, submitted to sonication and cultivation with probe-coated beads overnight at 4 °C. Lastly, RT-qPCR was employed to measure purified RNA complex.

### Luciferase reporter assay

Partial DNA sequences of SFN or LINC01128 containing wild-type (WT) or mutant (MUT) miR-383-5p binding sites were amplified by PCR and then cloned into a pmirGLO dual-luciferase plasmid (Promega, Madison, WI, USA) to produce SFN-WT, SFN-MUT, LINC01128-WT, and LINC01128-MUT reporter plasmids, which were separately co-transfected with miR-383-5p mimics or NC mimics into HeLa or SiHa cells. Finally, luciferase activities were evaluated through the dual luciferase reporter assay system (Promega).

### Statistical analysis

Results were presented as mean ± SD of three independent experiments at least. Statistical analyses were imported into SPSS 17.0 (SPSS Inc., Chicago, IL, USA). Student’s t-test or one-way ANOVA was employed for analysis of differences. *P* < 0.05 had statistically significant.

## Results

### The high expression of SFN promotes the cellular process of CC cells

**Fig. 1:**
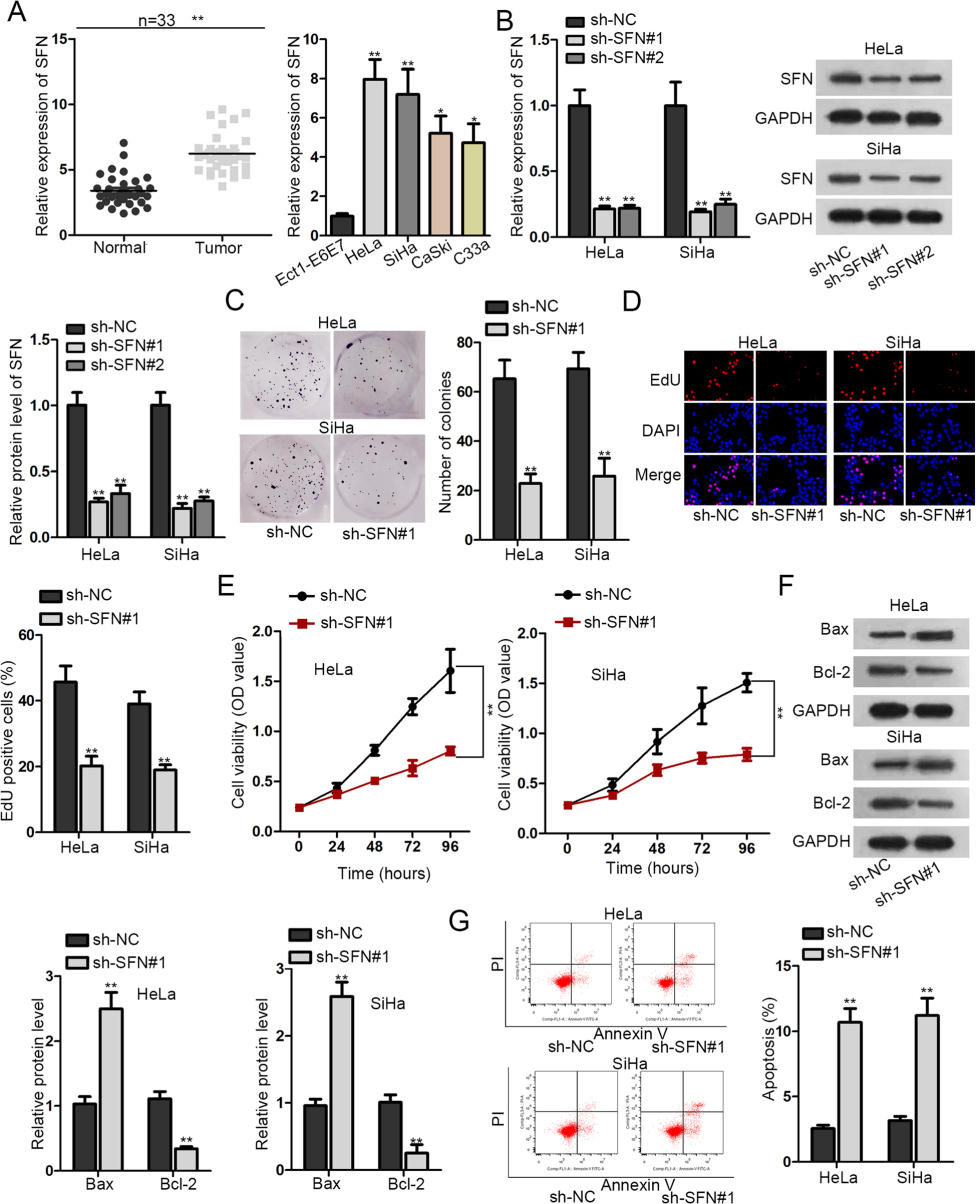
The high expression of SFN promotes the cellular process of CC cells. **a**. RT-qPCR was used to detect SFN expression in normal tissues and CC tissues, as well as normal cell (Ect1-E6E7) and CC cells (HeLa, SiHa, CaSki, C33a). **b**. The expression of SFN was assessed by RT-qPCR and western blot assays after transfecting with sh-SFN#1 and sh-SFN#2 in HeLa and SiHa cell lines. **c**-**e**. Colony formation assay, EdU stained assay and CCK-8 assay were performed to estimate cell proliferation. **f**. Western blot analysis of apoptosis related protein (Bax and Bcl-2) levels. **g**. Flow cytometry analysis was used to determine cell apoptotic ability. *p < 0.05, **p < 0.01

It was spotted in TCGA database that SFN expression was dramatically upregulated in cervical squamous cell carcinoma and endocervical adenocarcinoma (Additional file 1: Figure S1A). In subsequence, we examined SFN expression in normal tissues and CC tissues, as well as normal cell (Ect1-E6E7) and CC cell lines (HeLa, SiHa, CaSki and C33a) by RT-qPCR. The results showed that the expression of SFN was significantly higher in tumor tissues than normal tissues, as well as in CC cells than normal cell (Fig. 1a). Moreover, the correlation between SFN expression and clinicopathological features of CC patients was explored. As shown in Table 1, high expression of SFN is significantly correlated with FIGO stage and differentiation. Owing to higher expression in HeLa and SiHa cell lines than other cell lines, HeLa and SiHa cell lines were used for following experiments. Secondly, we verified the interference efficiency of SFN in HeLa and SiHa cell lines. The results showed that SFN expression was declined after SFN knockdown (Fig. 1b). Sh-SFN#1 presented the most obvious knockdown efficiency, so we chose it for the next function experiments. HeLa and SiHa cell lines were transfected with sh-NC and sh-SFN#1. We conducted colony formation assay, EdU stained assay and CCK-8 assay to test cell proliferation capacity. These results all indicated the descending proliferation ability of CC cells by depleting SFN (Fig. 1c-e, Additional file 1: Figure S1B). And then to measure the apoptosis capability of CC cells, we detected the relative level of apoptosis correlated protein (Bax and Bcl-2) after SFN being knocked down. It discovered that Bax protein level was ascended, whereas Bcl-2 protein level was decreased (Fig. 1f). Besides, flow cytometry analysis showed that SFN interference enhanced cell apoptotic ability (Fig. 1g). Moreover, the number of migrated and invaded cells was reduced by SFN deficiency in HeLa and SiHa cell lines (Additional file 1: Figure S1C). Finally, shortage of SFN was revealed to downregulate the protein level of N-cadherin, Vimentin, ZEB1, Slug, Twist and Snail as well as upregulate E-cadherin, implying that EMT process was hindered (Additional file 1: Figure S1D). Altogether, SFN was highly expressed in CC tissues and cells, and acted as an oncogene in CC progression.

**Table 1:** Correlation between SFN expression and clinicopathological features. (*n* = 33)

Variable	SFN Expression		*P*-value
	low	high	
Age			
< 45	3	5	0.688
> =45	13	12	
Tumor size			
< 4 cm	7	6	0.728
> 4 cm	9	11	
FIGO stage			
I/II	10	3	0.013^*^
III/IV	6	14	
Differentiation			
Well	11	4	0.015^*^
Poorly	5	13	
Histology			
Squamous	4	6	0.708
Adenocarcinoma	12	11	
Lymph node metastasis			
Negative	11	10	0.721
Positive	5	7	

Low/high by the sample median

Pearson χ^2^ test

*P < 0.05 was considered to be statistically significant

### SFN functions as a target gene of miR-383-5p

**Fig. 2:**
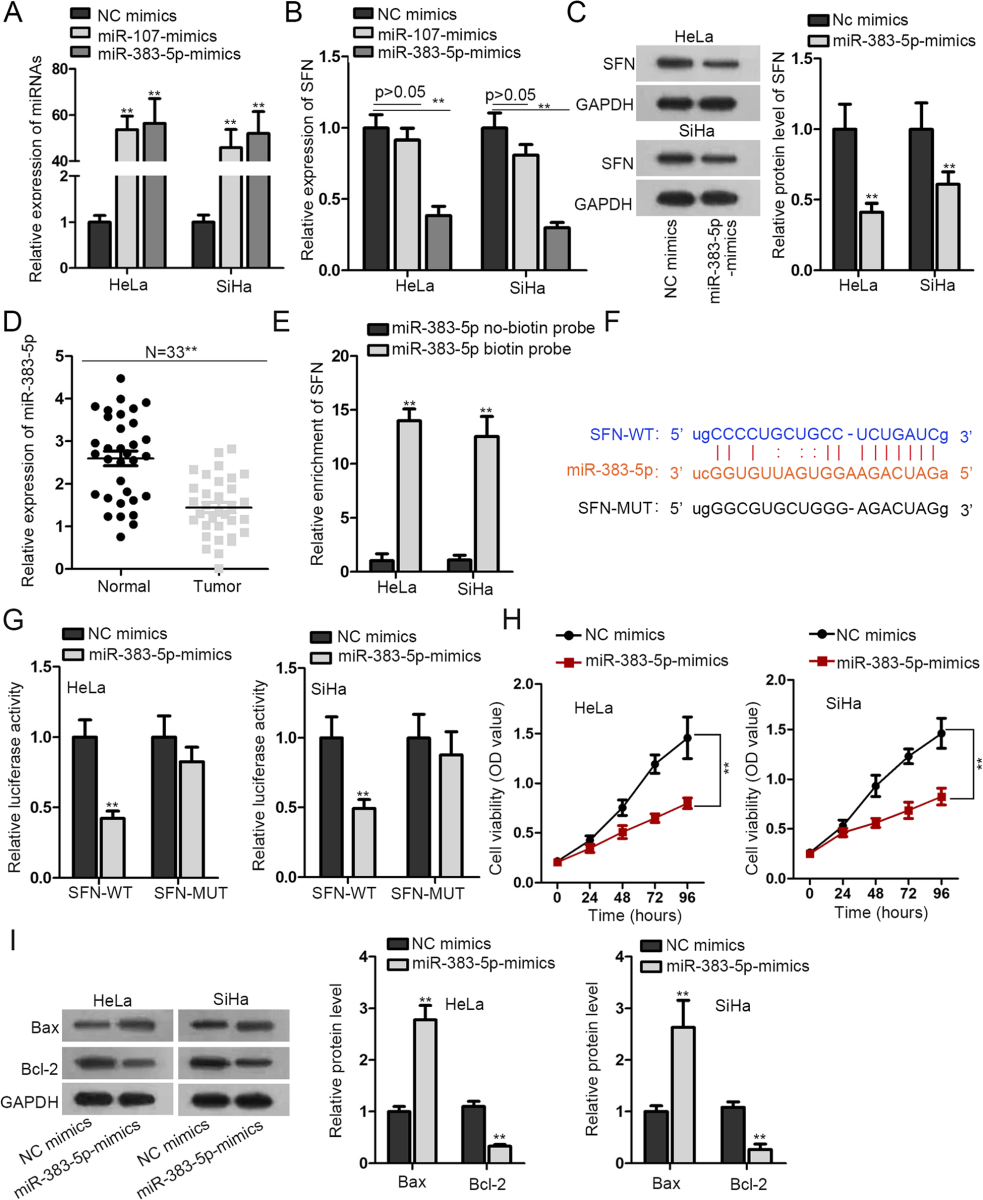
SFN functions as a target gene of miR-383-5p. **a**. The interference efficiency of miR-107 and miR-383-5p were estimated by RT-qPCR in HeLa and SiHa cells. **b**. The expression of SFN was determined via RT-qPCR after overexpressing miR-107 and miR-383-5p. **c**. The protein level of SFN was detected by western blot. **d**. miR-383-5p expression in CC tissues and adjacent normal tissues was measured by RT-qPCR. **e**. RNA pull down assay examined the binding association of LINC01128 and miR-383-5p by which precipitated with miR-383-5p no-biotin probe or miR-383-5p biotin probe. **f**. The predicting binding sites between LINC01128 and miR-383-5p were obtained from starBase website. **g**. Luciferase reporter assay was implemented to test the interaction relationship between LINC01128 and miR-383-5p. **h**. HeLa and SiHa cells were transfecting with miR-383-5p-mimics. Cell proliferation ability was analyzed by CCK-8 assay. **i**. Bax and Bcl-2 protein levels were checked by western blot. **p < 0.01

Primarily, to find out miRNA that could bind to SFN, we adopted Starbase to search for candidate miRNAs. Through the CLIP Data: strict stringency (> = 5) and the Degradome Data: medium stringency two conditions (> = 2), we screened out miR-107 and miR-383-5p. In addition, we detected the overexpression efficiency of miR-107 and miR-383-5p in HeLa and SiHa cell lines. The results revealed that miR-107 and miR-383-5p expression were both increased after overexpressing miR-107 and miR-383-5p (Fig. 2a). Next, we examined the SFN expression after transfection NC mimics, miR-107 mimics and miR-383-5p mimics in HeLa and SiHa cell lines. The results manifested that only overexpressing miR-383-5p could downregulate SFN. Therefore, miR-383-5p was selected as the upstream gene of SFN (Fig. 2b). Additionally, we evaluated the SFN protein level after miR-383-5p overexpression in HeLa and SiHa cell lines, and SFN protein level was reduced after increasing miR-383-5p expression (Fig. 2c). Besides, miR-383-5p was analyzed to be conspicuously downregulated in CC tissues (Fig. 2d). And then, we carried out RNA pull down assay to verify the combination relation between SFN and miR-383-5p. The results demonstrated the expression of SFN was enriched in miR-383-5p biotin probe group (Fig. 2e). To move on, the underlying binding sites were probed between SFN and miR-383-5p (Fig. 2f). Consistently, we made luciferase reporter experiments so as to attest the interaction relationship between SFN and miR-383-5p. It was observed that the luciferase activity of wild type SFN (SFN-WT) was eminently declined by miR-383-5p overexpression, while there was no change of mutant type SFN (SFN-MUT) group (Fig. 2g). Apart from that, we performed CCK-8 assay to reflect cell proliferation capacity after overexpressing miR-383-5p. The data indicated cell proliferation ability was declined via increasing miR-383-5p (Fig. 2h). On the contrary, cell apoptotic capability was promoted. It was concluded from the results that the protein level of Bax was elevated in miR-383-5p mimics-transfected cells (Fig. 2i). In addition, spheroid formation assay indicated that enforced expression of miR-383-5p suppressed spheroid formation (Additional file 1: Figure S1E).

### LINC01128 functions as miR-383-5p molecular sponge

**Fig. 3:**
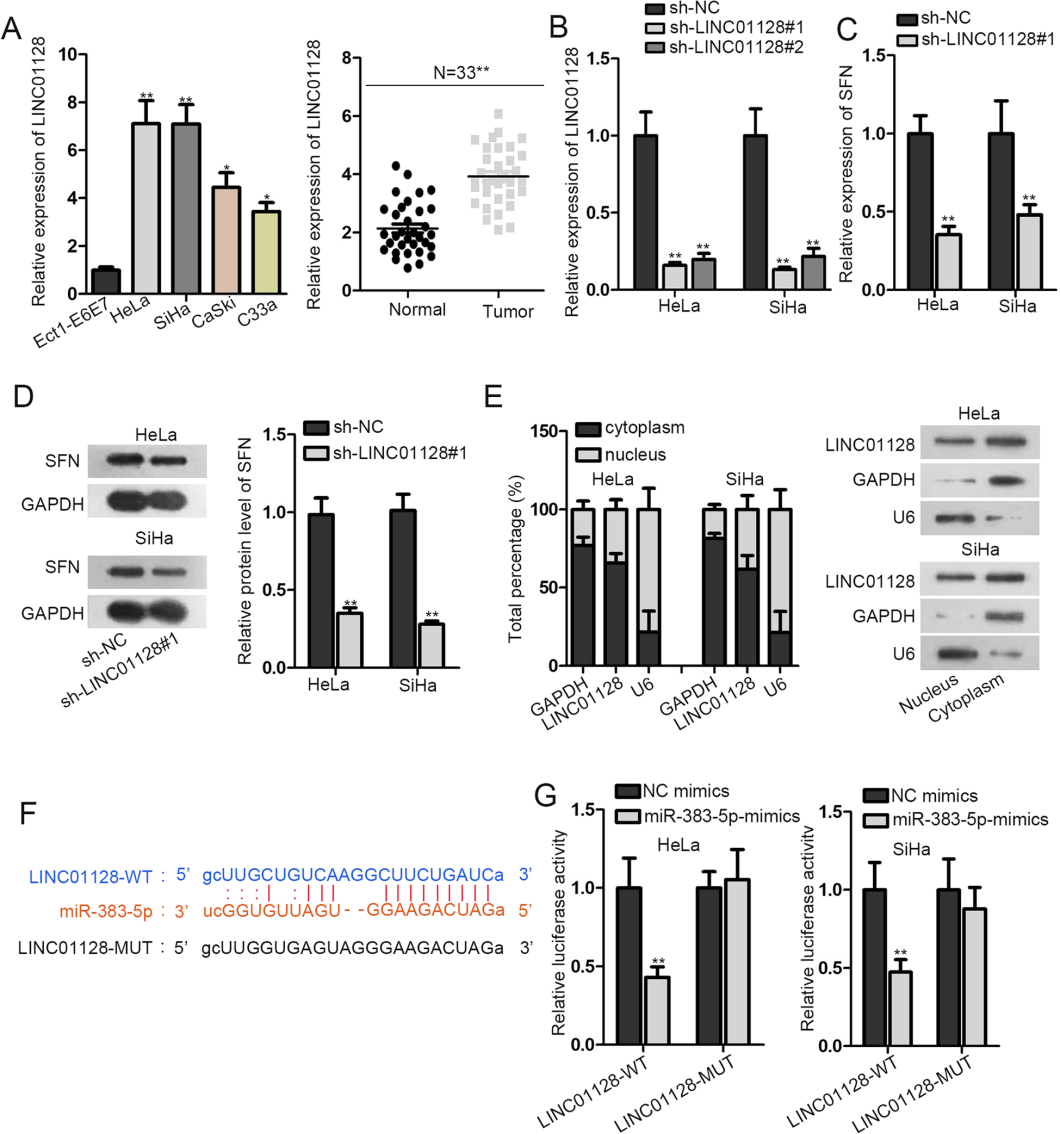
LINC01128 functions as miR-383-5p molecular sponge. **a**. RT-qPCR examined LINC01128 expression in CC tissues and adjacent normal tissues. The expression of LINC01128 was tested in normal cell line and CC cell lines by RT-qPCR. **b**. The knockdown efficiency of LINC01128 was examined by RT-qPCR in CC cells transfected with sh-LINC01128–1 and sh-LINC01128–2. **c**-**d**. The expression and protein level of SFN were determined after LINC01128 interference by RT-qPCR and western blot. **e**. Subcellular fractionation and northern blot assays suggested LINC01128 was predominantly located in the cytoplasm of CC cells. **f**. The complementary base pairing between miR-324-3p and LINC01128 were acquired from starBase website. **g**. Luciferase reporter assay was implemented to attest the interaction correlation of miR-324-3p and LINC01128. *p < 0.05, **p < 0.01

Through Starbase, we found that LINC01128 could bind with miR-383-5p. Hence, LINC01128 was chosen to be investigated in the following experiments. To begin with, the expression of LINC01128 was figured out to be remarkably upregulated in CC tissues. Then we detected the expression of LINC01128 in normal cell (Ect1-E6E7) and CC cell lines (HeLa, SiHa, CaSki and C33a). We found LINC01128 expression was prominently high in HeLa and SiHa cells (Fig. 3a). After that, we examined the knockdown efficiency of LINC01128. HeLa and SiHa cells were silenced by two shRNAs targeting LINC01128 (sh-LINC01128#1 and sh-LINC01128#2). The results indicated that LINC01128 expression was remarkably decreased after intervening LINC01128 (Fig. 3b). Sh-LINC01128#1 exhibited the best knockdown efficiency, so it was selected for the following assay. In subsequence, the SFN mRNA and protein levels after LINC01128 knockdown were tested. We observed SFN mRNA and protein levels were both clearly descended (Fig. 3c-d). Moreover, to define the location of LINC01128, we did subcellular fractionation experiment. We discovered that LINC01128 was mostly scattered in the cytoplasmic (Fig. 3e). Furthermore, the predicted binding sites between LINC01128 and miR-383-5p were found (Fig. 3f). To continue, we carried out the luciferase reporter experiment by co-transfecting LINC01128-WT/LINC01128-MUT, NC mimics and miR-383-5p mimics into HeLa and SiHa cells. The results revealed that the fluorescence activity of LINC01128-WT was noticeably declined, while that of LINC01128-MUT was almost unchanged (Fig. 3g). These results unveiled that the binding and interaction relationship between LINC01128 and miR-383-5p.

### LINC01128 accelerates proliferation and restrains apoptosis of CC cells by sponging miR-383-5p and targeting SFN

**Fig. 4:**
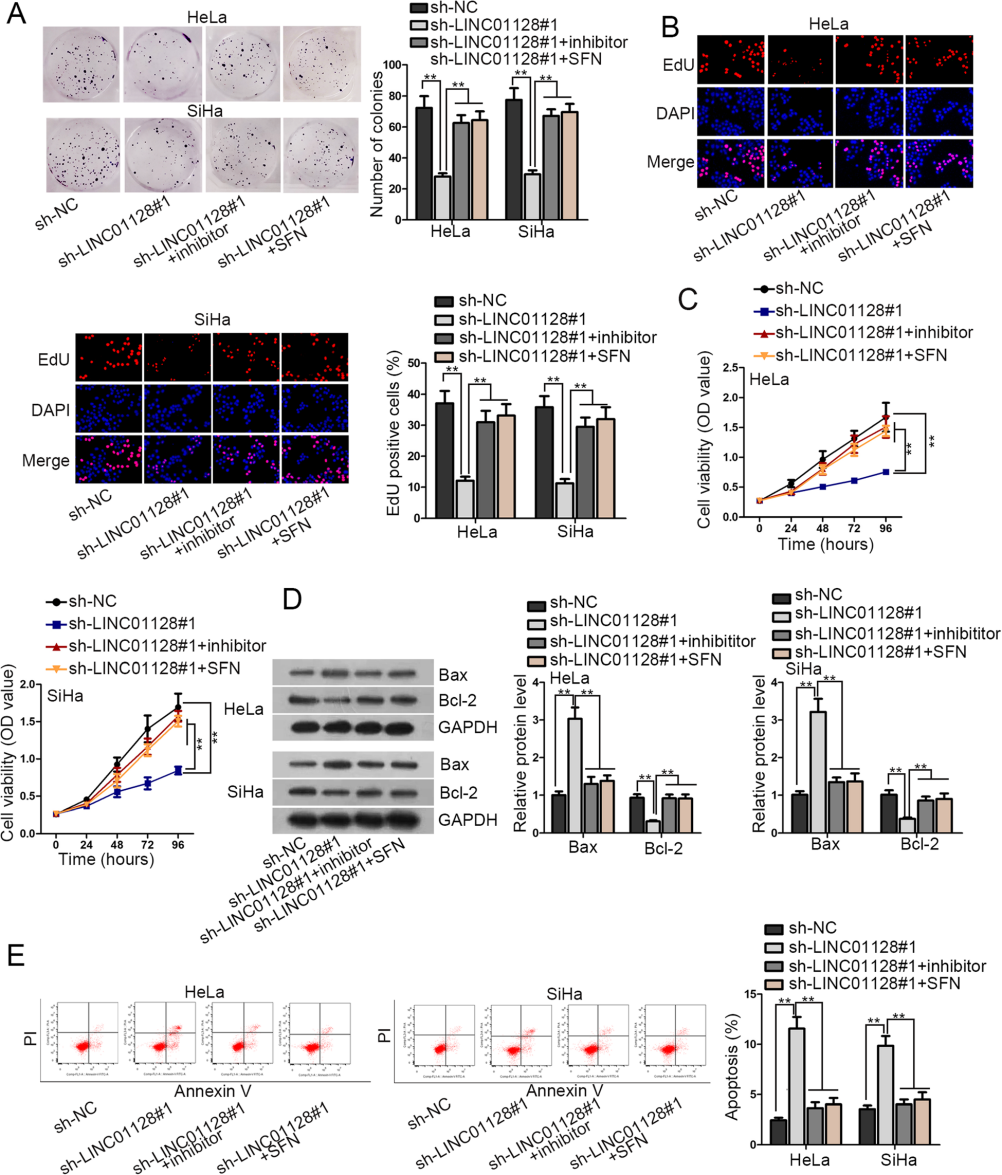
LINC01128 accelerates proliferation and restrains apoptosis of CC cells by sponging miR-383-5p and targeting SFN. **a**-**c**. HeLa and SiHa cells were co-transfected with sh-LINC01128#1and miR-383-5p inhibitor or pcDNA3.1/SFN. Colony formation assay, EdU stained assay and CCK-8 assay were to determine cell proliferation abilities. **d**. Bax and Bcl-2 protein levels were checked by western blot. **e**. Flow cytometry analysis of cell apoptosis capacity in HeLa and SiHa cells. **p < 0.01

At last, functional rescue experiments were applied to confirm whether LINC01128 promoted the development of CC by targeting miR-383-5p/SFN axis. HeLa and SiHa cells were co-transfected with sh-LINC01128#1 and miR-383-5p inhibitor or pcDNA3.1/SFN. First of all, colony formation assay, EdU stained assay and CCK-8 assay demonstrated that cell proliferation ability was decreased after LINC01128 knockdown. This decrease was restored after interfering miR-383-5p, as well as SFN overexpression (Fig. 4a-c). Next, western blot analysis indicated that Bcl-2 protein level was decreased after LINC01128 knockdown, while was recovered after miR-383-5p interference or overexpressing SFN (Fig. 4d). Furthermore, flow cytometry analysis showed cell apoptosis ability was enhanced by depleting LINC01128, and was further repressed via intervening miR-383-5p or increasing SFN (Fig. 4e). Moreover, inhibition of miR-383-5p or enforced expression of SFN could compensate for the suppression in cell migration, invasion and EMT process induced by LINC01128 silencing. (Additional file 2: Figure S2A-B). At last, 3D spheroid formation assay revealed that the restrained spheroid formation triggered by LINC01128 shortage could be recovered by the transfection of miR-383-5p inhibitor or pcDNA3.1/SFN. (Additional file 2: Figure S2C).

## Discussion

CC is one of the most common gynecological malignancies with significant morbidity and mortality in women. It is often reported that there is an urgent need to screen biomarkers that can be used for clinical diagnosis and treatment of CC patients due to the reasons of delayed diagnosis and poor treatment effect. In present study, we detected the expressions of SFN in CC tumor tissues and cells were much stronger. In addition, interfering SFN dramatically inhibited cells proliferation, migration, invasion and EMT process as well as promoted cells apoptosis. Thus, we inferred that SFN acted as an oncogene in CC cells.

Then, miR-383-5p was identified as the upstream gene of SFN. In the meantime, reports have shown inhibitory effects of miR-383-5p in adenocarcinoma [27, 28]. Besides, miR-383-5p acted as a tumor suppressor to get involved in the ceRNA mechanism [29, 30]. miR-383-5p was quantified to be downregulated in CC tissues. Additionally, we found that SFN was enabled to bind with miR-383-5p and be suppressed by miR-383-5p overexpression. Functionally, miR-383-5p overexpression could induce an inhibition in cell proliferation of CC cells.

The dysregulation of lncRNA was identified to be associated with the development of disease [31]. Besides that, lncRNA plays an extremely significant role in various biological processes such as proliferation and apoptosis. Since several lncRNAs were found to bind and isolate miRNAs and block the role of miRNAs, we have speculated the presence of lncRNAs that combine and interdict with miR-383-5p and play a carcinogenic role in CC. And LINC01128 was screened out from Starbase to bind to miR-383-5p. Nevertheless, literature concerning LINC01128 is very limited. In this work, it was spotted that LINC01128 exhibited conspicuous high expression in CC tissues and cells. Moreover, LINC01128 shortage could suppress SFN level. Next, we discovered the location of LINC01128 in cytoplasm through nuclear cytoplasm separation experiment. Besides that, LINC01128 was verified to bind to miR-383-5p. Finally, it was proved that LINC01128 enhances the malignancy of CC dependent on miR-383-5p and SFN.

The major limitation of our study lies in the absence of in vivo assays, through which we could observe the influences of SFN on CC cells growth and metastasis. We would carry out in vivo assays in further investigation. Anchored in the above evidences, it could be concluded that LINC01128 poses promotional influences on cell proliferation, migration, invasion and EMT process in CC via sponging miR-383-5p and upregulating SFN. In other words, the LINC01128/miR-383-5p/SFN axis was conducive to enhance the CC progression.

## Conclusion

In summary, the availability of miR-383-5p was antagonized by LINC01128 for the SFN-induced cell proliferation, migration, invasion and EMT process in cervical cancer, shedding a new light into understanding CC.

## Supplementary information

**Additional file 1:** **Figure S1.** A. The expression of SFN in cervical squamous cell carcinoma and endocervical adenocarcinoma (CESC) tissue samples was found in TCGA database. B. The number of colonies in sh-SFN#1-transfected cells was unveiled by colony formation assay. C. Transwell assay reflected cell migration and invasion in sh-SFN#1-transfected cells. D. Western blot assay measured the level of EMT process-related proteins in transfected cells. E. Spheroid formation assay reflected the spheroid formation in miR-324-3p mimics-transfected cells. *p < 0.05, **p < 0.01.

**Additional file 2:** **Figure S2.** A. Transwell assay evaluated cell migration and invasion after cells being knocked down with LINC01128. B. The level of EMT process-related proteins in transfected cells was quantified by western blot assay. C. The spheroid formation in HeLa and SiHa cells co-transfected with sh-LINC01128#1 and miR-383-5p inhibitor or pcDNA3.1/SFN was measured by spheroid formation assay. **p < 0.01.

## Data Availability

Not applicable.

## Abbreviations

CC: Cervical cancer
CCK-8: Cell counting kit-8
ceRNA: Endogenous competitive RNA
EdU: 5-ethynyl-2′-deoxyuridine
lncRNA: long non-coding RNA
miRNA: microRNA
mRNA: messenger-RNA
Mut: Mutant type
RT-qPCR: Real-time quantitative PCR
SFN: Stratifin
Wt: Wide type

## References

[1] Morris E, Roett MA (2015). Genital cancers in women: cervical Cancer. FP Essent.

[2] Pimple S, Mishra G, Shastri S (2016). Global strategies for cervical cancer prevention. Curr Opin Obstet Gynecol.

[3] Wentzensen N, Schiffman M (2018). Accelerating cervical cancer control and prevention. Lancet Public Health.

[4] Stewart E (2018). Identification of Therapeutic Targets in Rhabdomyosarcoma through Integrated Genomic, Epigenomic, and Proteomic Analyses. Cancer Cell.

[5] Vu M (2018). Cervical cancer worldwide. Curr Probl Cancer.

[6] Shiba-Ishii A (2015). Stratifin accelerates progression of lung adenocarcinoma at an early stage. Mol Cancer.

[7] Hu Y, et al. Expression profile and prognostic value of SFN in human ovarian cancer. Biosci Rep. 2019;39(5). 10.1042/BSR20190100. 10.1042/BSR20190100 PMC6499453 30926680

[8] Ren HZ (2010). Reduced stratifin expression can serve as an independent prognostic factor for poor survival in patients with esophageal squamous cell carcinoma. Dig Dis Sci.

[9] Bu Y (2018). A PERK-miR-211 axis suppresses circadian regulators and protein synthesis to promote cancer cell survival. Nat Cell Biol.

[10] Wu MZ (2017). miR-25/93 mediates hypoxia-induced immunosuppression by repressing cGAS. Nat Cell Biol.

[11] Celia-Terrassa T (2017). Normal and cancerous mammary stem cells evade interferon-induced constraint through the miR-199a-LCOR axis. Nat Cell Biol.

[12] Yuan JH (2011). The histone deacetylase 4/SP1/microrna-200a regulatory network contributes to aberrant histone acetylation in hepatocellular carcinoma. Hepatology.

[13] Hosseini ES (2017). Dysregulated expression of long noncoding RNAs in gynecologic cancers. Mol Cancer.

[14] Yang Y (2017). Long non-coding RNAs in colorectal Cancer: progression and future directions. J Cancer.

[15] Zhu XT (2016). Long noncoding RNA glypican 3 (GPC3) antisense transcript 1 promotes hepatocellular carcinoma progression via epigenetically activating GPC3. FEBS J.

[16] Hu X (2014). A functional genomic approach identifies FAL1 as an oncogenic long noncoding RNA that associates with BMI1 and represses p21 expression in cancer. Cancer Cell.

[17] Lin A (2017). The LINK-A lncRNA interacts with PtdIns (3,4,5) P3 to hyperactivate AKT and confer resistance to AKT inhibitors. Nat Cell Biol.

[18] Yuan JH (2017). The MBNL3 splicing factor promotes hepatocellular carcinoma by increasing PXN expression through the alternative splicing of lncRNA-PXN-AS1. Nat Cell Biol.

[19] Uszczynska-Ratajczak B (2018). Towards a complete map of the human long non-coding RNA transcriptome. Nat Rev Genet.

[20] Pop S (2018). Long non-coding RNAs in brain tumours: focus on recent epigenetic findings in glioma. J Cell Mol Med.

[21] Cesana M (2011). A long noncoding RNA controls muscle differentiation by functioning as a competing endogenous RNA. Cell.

[22] Wang H (2017). STAT3-mediated upregulation of lncRNA HOXD-AS1 as a ceRNA facilitates liver cancer metastasis by regulating SOX4. Mol Cancer.

[23] Yuan JH (2014). A long noncoding RNA activated by TGF-beta promotes the invasion-metastasis cascade in hepatocellular carcinoma. Cancer Cell.

[24] Salmena L (2011). A ceRNA hypothesis: the Rosetta stone of a hidden RNA language?. Cell.

[25] Wang Y (2018). Long noncoding RNA DANCR, working as a competitive endogenous RNA, promotes ROCK1-mediated proliferation and metastasis via decoying of miR-335-5p and miR-1972 in osteosarcoma. Mol Cancer.

[26] Wang Y, Zhang X (2008). Characterization of a novel portal protein from deep-sea thermophilic bacteriophage GVE2. Gene.

[27] Zhao S (2017). MicroRNA-383-5p acts as a prognostic marker and inhibitor of cell proliferation in lung adenocarcinoma by cancerous inhibitor of protein phosphatase 2A. Oncol Lett.

[28] Li J (2018). MicroRNA-383 acts as a tumor suppressor in colorectal cancer by modulating CREPT/RPRD1B expression. Mol Carcinog.

[29] Jiang J (2019). Up-regulation of miR-383-5p suppresses proliferation and enhances chemosensitivity in ovarian cancer cells by targeting TRIM27. Biomed Pharmacother.

[30] Tu Chaoyong, Chen Wei, Wang Shuqian, Tan Wei, Guo Jingqiang, Shao Chuxiao, Wang Weilin (2019). MicroRNA‐383 inhibits doxorubicin resistance in hepatocellular carcinoma by targeting eukaryotic translation initiation factor 5A2. Journal of Cellular and Molecular Medicine.

[31] Yu CY, Chuang CY, Kuo HC (2018). Trans-spliced long non-coding RNA: an emerging regulator of pluripotency. Cell Mol Life Sci.

