# Supplementary figures and images for: LINC01128 expedites cervical cancer progression by regulating miR-383-5p/SFN axis
Source: BMC Cancer. 2019 Nov 28;19:1157. doi: 10.1186/s12885-019-6326-5 (PMC6883532; doi:10.1186/s12885-019-6326-5)

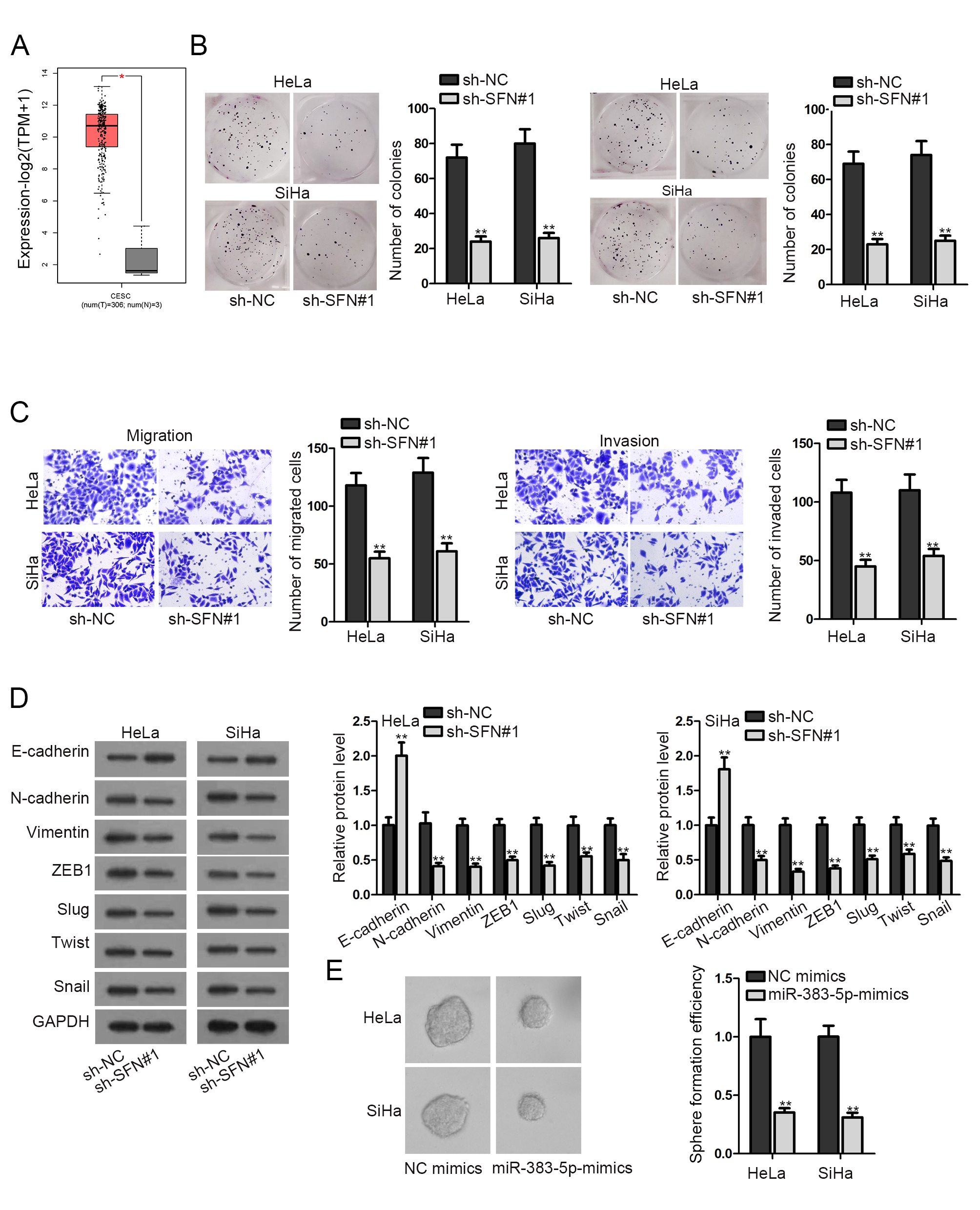

Supplement: Supplementary file 1 — Additional file 1: Figure S1. A. The expression of SFN in cervical squamous cell carcinoma and endocervical adenocarcinoma (CESC) tissue samples was found in TCGA database. B. The number of colonies in sh-SFN#1-transfected cells was unveiled by colony formation assay. C. Transwell assay reflected cell migration and invasion in sh-SFN#1-transfected cells. D. Western blot assay measured the level of EMT process-related proteins in transfected cells. E. Spheroid formation assay reflected the spheroid formation in miR-324-3p mimics-transfected cells. *p < 0.05, **p < 0.01. [file 12885_2019_6326_MOESM1_ESM.tif]

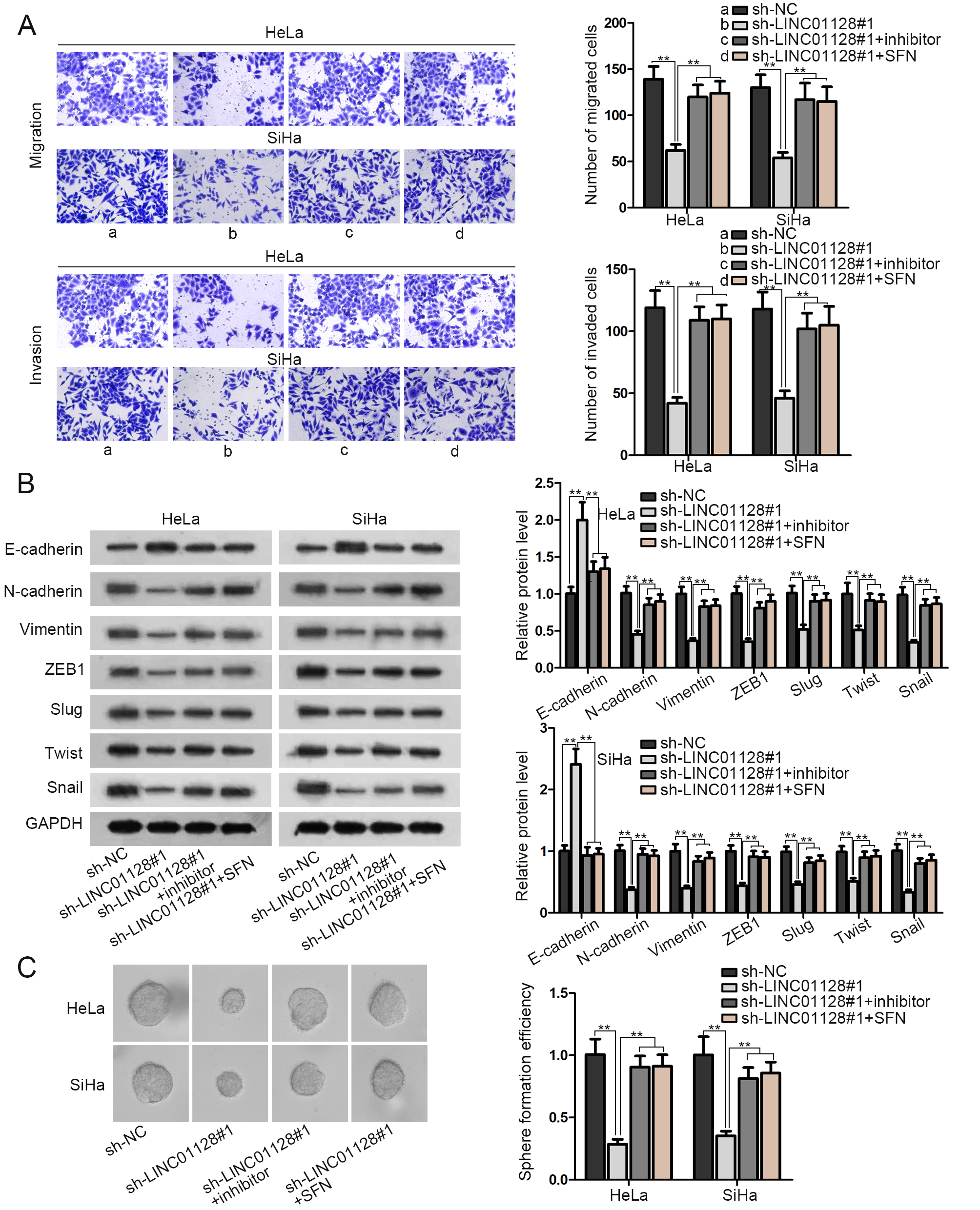

Supplement: Supplementary file 2 — Additional file 2: Figure S2. A. Transwell assay evaluated cell migration and invasion after cells being knocked down with LINC01128. B. The level of EMT process-related proteins in transfected cells was quantified by western blot assay. C. The spheroid formation in HeLa and SiHa cells co-transfected with sh-LINC01128#1 and miR-383-5p inhibitor or pcDNA3.1/SFN was measured by spheroid formation assay. **p < 0.01. [file 12885_2019_6326_MOESM2_ESM.tif]
